# Effect of Chopped ZrO_2_ Fiber Content on the Microstructure and Properties of CaO-Based Integral Ceramic Mold

**DOI:** 10.3390/ma13235398

**Published:** 2020-11-27

**Authors:** Qiang Yang, Fu Wang, Dichen Li

**Affiliations:** State Key Laboratory for Manufacturing System Engineering, School of Mechanical Engineering, Xi’an Jiaotong University, Xi’an 710049, China; yangqiang@stu.xjtu.edu.cn (Q.Y.); xjtudcli@sina.com (D.L.)

**Keywords:** ZrO_2_ fiber, calcium oxide, gel-casting, microstructure, properties

## Abstract

A chopped ZrO_2_ fiber (ZrO_2__(f)_) reinforced CaO-based integral ceramic mold was successfully fabricated by stereolithography (SLA) and tert-butyl alcohol (TBA)-based gel-casting, and the effect of chopped ZrO_2__(f)_ content on properties of the ceramic mold was investigated. The results show that the ZrO_2__(f)_ content had a significant effect on the viscosity of CaO-based ceramic slurry, which directly affects the filling ability of slurry in complex structures of the integral mold. The tiny structures of the ceramic mold cannot be filled completely with a ZrO_2__(f)_ content exceeding 3 vol %. The sample fabricated with 3 vol % fiber content showed a harmonious microstructure and exhibited an excellent comprehensive performance with 25 °C bending strength of 22.88 MPa, an 1200 °C bending strength of 15.74 MPa, a 1200 °C deflection of 0.86 mm, and a sintering shrinkage of 0.40%, which can meet the requirements of casting very well.

## 1. Introduction

CaO has long been considered an attractive refractory for application in metallurgical and ceramics industries because of the global abundant sources of high-purity limestone, as well as its advantages of high melting temperature, low vapor pressure, and excellent thermodynamic stability [1,2,3,4,5]. In recent years, CaO was studied as a potential ceramic core material for investment casting due to the reaction-resistance to molten active alloy [6,7,8], easy dissolution compared to common ceramic core materials, such as fused silica or alumina [9,10,11], and its approximating the thermal expansion coefficient of superalloys [12]. However, in spite of these primary advantageous properties, the application of CaO is inhibited due to its hydration susceptibility in atmospheric moisture [2,3,4,5,13,14], which increased the difficultly of fabricating CaO ceramics as well. Most existing methods for forming CaO ceramic, such as hot-pressing [1] and cold-pressing [3,4,5], can only fabricate simple architectures, which makes them untenable for generating complex ceramic structures.

The rapid integral fabrication technique (RIFT), based on stereolithography (SLA) and non-aqueous gel-casting technology, provides a new approach for fabricating complex ceramic components, especially the ceramic molds of hollow turbine blades [15,16,17,18]. Tert-butyl alcohol (TBA) is selected as solvent, on the one hand because it can effectively avoid the hydrolysis of CaO powder, and on the other hand because of its high saturation vapor pressure and low surface tension force: the green body can be easily dried with a small shrinkage and deformation [19]. In order to meet the requirement of investment casting, the ceramic mold must has a good performance in mechanical properties and no structure defects, such as cracks, severe deflection, or shrinkage deformation. However, cracking often occurs during the pre-sintering of the RIFT process. In this stage, the integrity of the ceramic mold can be only maintained by the particle packing effects because the gel networks were burnt off. Therefore, the slender structures may be broken by gravity and the thin-wall structures also may be broken by the stress caused by pyrolysis of the resin prototype. Moreover, the integral ceramic mold always has slightly poor mechanical properties, a lager deflection, and sintering shrinkage. The unacceptable drawbacks for investment casting of ceramic mold can be overcome by incorporating particulate, whisker, or fiber into the base materials to form ceramic matrix composites [20,21]. Especially using chopped fibers, the ceramic can not only be strengthened in isotropic properties due to the homogenous distribution, but also the ceramic mold with complex structures can be formed by conventional manufacturing techniques, such as slip casting or gel-casting [22,23].

In this work, the chopped ZrO_2 (f)_ was selected to reinforce the CaO-based integral ceramic mold due to its high melting point, excellent strength and toughness, low thermal conductivity, super corrosion, and oxidation resistance [24]. In addition, the chopped ZrO_2 (f)_ can be well retained during the sintering of the CaO matrix, because it does not react with the CaO matrix at the lower sintering temperature unlike the silica or alumina fibers. The CaO-based integral ceramic molds are fabricated by the RIFT technique with the TBA-based gel-casting. The effects of chopped ZrO_2 (f)_ content on the viscosities and filling ability of the slurries, the microstructures, mechanical properties, deflection, and sintering shrinkage of ceramic mold were investigated, and the reinforcing mechanism of chopped ZrO_2 (f)_ reinforced porous CaO ceramic mold is also discussed.

## 2. Material and Methods

### 2.1. Raw Materials

**Figure 1:**
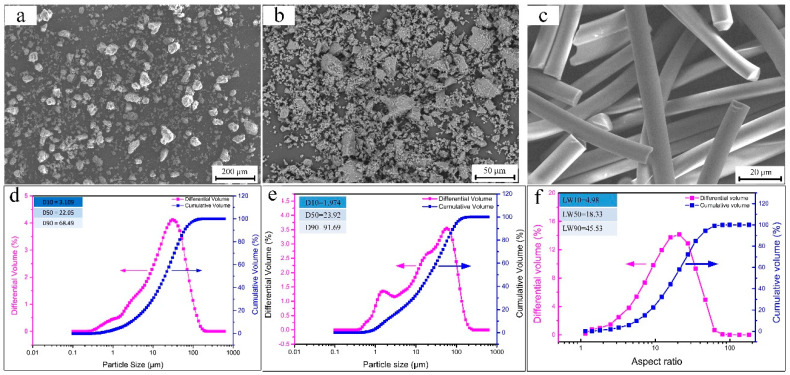
(**a**) SEM micrograph of the CaO powders, (**b**) SEM micrograph of the MgO powders, (**c**) SEM micrograph of the ZrO_2 (f)_, (**d**) particle size distribution of the CaO powders, (**e**) particle size distribution of the MgO powders, (**f**) aspect ratio of ZrO_2 (f)_.

Commercial CaO powders (purity ≥99%, Xing Tai special ceramics Co. Ltd., Xi’an, China), commercial fused MgO powders (Henghai magnesium industry Co., Ltd., Lianyungang, China), and chopped ZrO_2_ fiber (mean diameter and length are 5~8 μm and 200 μm, respectively, purity ≥99%, Shandong Huolong Ceramic Fiber Co., Ltd., Jinan, China) were used as the raw materials. Zirconium powder (AR, Western BaoDe Technologies Co., Ltd., Xi’an, China) with a mean particle diameter of 5 μm was used as the sintering additive. The morphology of the CaO powders, MgO powders, and ZrO_2 (f)_, the particle size distribution of the CaO powders, MgO powders, and the aspect ratio of ZrO_2 (f)_ are shown in Figure 1.

### 2.2. Experimental Procedure

**Figure 2:**
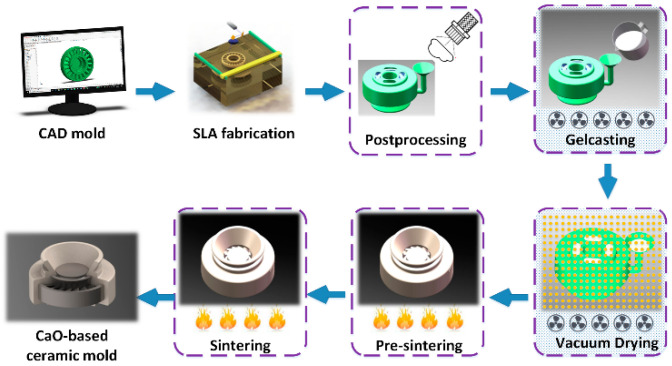
Fabrication flow chart of the CaO-based integral ceramic mold [18].

The rapid integral fabrication technique was applied to fabricate the CaO-based ceramic samples and molds, the fabrication flow chart was shown in Figure 2. The premixed solution was prepared by dissolving N, N-dimethylacrylamide (DMAA) and N, N-methylene diacrylamide (MBAM) into the Tert-butyl alcohol (TBA) with the weight ratio of DMAA:MBAM:TBA = 24:2:100. 3 wt.% polyvinyl pyrrolidone (PVP K30) with respect to the total mass of powders was added into the premixed solution as dispersant. All the chemical reagents are of AR purity and provided by Sinopharm Chemical Reagent Ltd., Shanghai, China. Due to the serious agglomeration of the chopped zirconia fibers, the ZrO_2 (f)_ must be pre-dispersed before use. The ZrO_2 (f)_ needs to be added into absolute ethyl alcohol, subsequently placed in an ultrasonic disperser for 2–4 h, and dried finally. Then the appropriate amount of CaO, MgO, and Zirconium powders and a certain amount of processed ZrO_2 (f)_ were added into the premixed solution by mechanical stirring, followed by the ball milling for 50~60 min. The prepared slurries exhibited a good flow-ability and stability and were degased for more than 5 min. After adding an appropriate amount of N,N-dimethylaniline (DMA) as initiator and benzoyl peroxide (BPO) as catalyst, the mixed slurry was poured into the SLA resin mold fabricated by SPS600B Rapid Prototyping Machine (Shaanxi Hengtong Intelligent Machine Co., Ltd., Xi’an, China) using photosensitive resin (SPR 8981; Zheng bang Ltd., Zhuhai, China) under vacuum and vibration conditions. After the polymerization of the monomers, the green body was put into the vacuum drying oven (Taisite Instrument Co., Ltd., Tianjin, China) at 40 °C for 48 h. At last, an integral CaO-based ceramic mold was obtained after being sintered at 1400 °C and kept for 3 h [18]. According to our previous study [18], during the fabrication process, the optimal total solid loading (the solid loading of CaO powder, MgO powder, Zirconium powder, and ZrO_2 (f)_) of the slurry was fixed at 56 vol %, while the content of ZrO_2 (f)_ varied from 0 vol % to 4 vol %; the volume fraction is calculated based on the assumption that the packing of powder particles is compact. The component of the composite powders is shown in Table 1.

### 2.3. Characterization and Testing

The particle sizes of the powders were measured by the Laser Particle Sizer (BT-9300S, Better Instruments Co., Ltd., Dandong, China). The viscosity of CaO-based ceramic slurries was tested by the Digital rotational viscometer (SNB-3, Shanghai NiRun Intelligent Technology Co., Ltd., Shanghai, China). The structures of the samples and the integral ceramic molds were investigated by the micro X-ray imaging system (industrial CT) (Y. Cheetah, YXLON, Hamburg, Germany) with a scanning resolution of 50 μm. The phase composition was identified by X-ray diffraction (XRD) (X’Pert Protype, PANalytical BV, Almelo, The Netherlands). The microstructure and elemental composition of samples were investigated by the field emission scanning electron microscope (SEM) (SU-8010, Hitachi Ltd., Tokyo, Japan). The bending strengths of samples were tested by a three-point bending test machine (HSST-6003QP, Sinosteel Luoyang Institute of Refractories, Luoyang, China) with a span distance of 30 mm at a crosshead speed of 6 mm/min using samples of a nominal size of 4 mm × 10 mm × 60 mm. The apparent porosity of sintered bodies were measured by immersion method in absolute ethyl alcohol under vacuum using Archimedes’ principle. The sintering shrinkage of samples was calculated according to the following Equation (1).

(1) ε=L1−L2L1×100%

where *ε* is the shrinkage of sample, *L*_1_ is the length of sample before sintering, *L*_2_ is the length of sample after sintering.

## 3. Result and Discuss

### 3.1. Effect on Viscosity and Filling Ability of the Slurry

**Figure 3:**
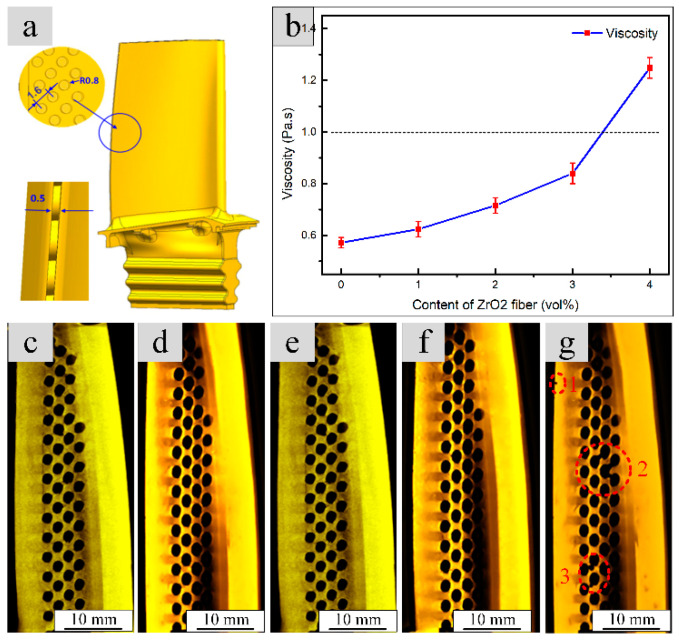
Effects of ZrO_2 (f)_ content on viscosity and filling ability of the slurries: (**a**) schematic of tiny and complex structures in the ceramic mold, (**b**) viscosity of slurries with different ZrO_2 (f)_ content, (**c**–**g**) CT images of different ZrO_2 (f)_ content: (**c**) 0 vol %, (**d**) 1 vol %, (**e**) 2 vol %, (**f**) 3 vol %, (**g**) 4 vol %.

It is always very difficult to fill the tiny and complex structures in the ceramic mold sufficiently during the fabrication process of the integral ceramic mold by RIFT process, as shown in Figure 3a. Some insufficient filled situations often occur in these tiny structures, which leads to the loss of the structural integrity of the mold and the failure of ceramic mold fabrication. The structural integrity of the ceramic mold is directly determined by the filling ability of the ceramic slurry, depending on the viscosity of the ceramic slurry. Generally speaking, in order to obtain a complete filling of the fine structure, the viscosity of the ceramic slurry is must less than 1 Pa s in the gel-casting process [17]. Since the viscosity of ceramic slurry is very sensitive to the content of ZrO_2 (f)_, it is very important to obtain an appropriate ZrO_2 (f)_ addition amount to realize the tiny structures duplication without defects.

Figure 3b–g shows the effect of ZrO_2 (f)_ content on viscosity and filling ability of the slurries. As can be seen from Figure 3b, the viscosity of slurries increased gradually with the increasing of ZrO_2 (f)_ content. When the ZrO_2 (f)_ content reaches 4 vol %, the viscosity exceed 1Pa·s, the limit of slurry viscosity in gel-casting process. As shown in Figure 3c–g, the tiny structures could be filled perfectly, when the ZrO_2 (f)_ content was not exceeding 3 vol %. However, some insufficient filled areas were found in the tiny structures as the ZrO_2 (f)_ content further increase to 4 vol %, as shown in Figure 3g (area 1, 2, 3). This phenomenon is mainly caused by the high viscosity (>1 Pa·s) of ceramic slurry, due to the addition of excessive fibers. Therefore, in order to guarantee that the tiny structures can be filled completely by the CaO-based ceramic slurry with a low viscosity, the ZrO_2 (f)_ content cannot exceed 3 vol % in the RIFT process to fabricate the ceramic mold with complex structures.

### 3.2. Microstructure and Phase Analysis

**Figure 4:**
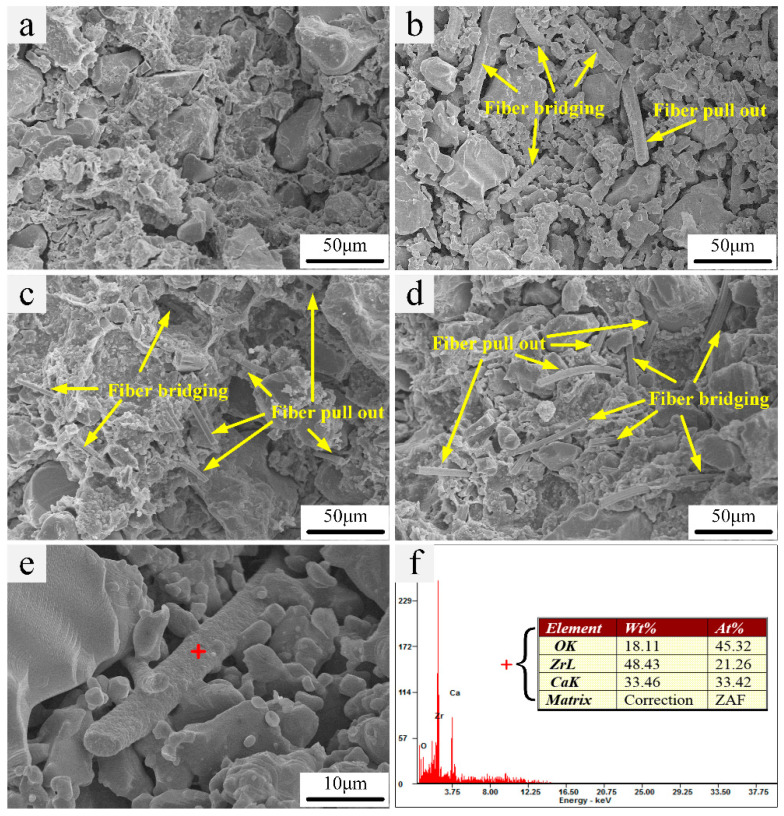
SEM micrographs of fracture surface with different ZrO_2 (f)_ content: (**a**) 1 vol %, (**b**) 2 vol %, (**c**) 3 vol %, (**d**) 4 vol %, (**e**,**f**) EDS of ZrO_2 (f)_ interface.

Figure 4a–d shows the fracture surface of the samples fabricated with different ZrO_2__(f)_ additions after sintered at 1400 °C for 3 h. As shown in Figure 4a–c, the ZrO_2 (f)_ are randomly arranged and homogeneously distributed in the CaO ceramic matrix when the content is not exceeding 3 vol %; whereas, the ZrO_2 (f)_ are likely to distribute in fasciculate when the content is up to 4 vol %, as shown in Figure 4d. The heterogeneous distribution of ZrO_2 (f)_ is caused by the insufficient dispersion of excess fiber in slurry. It can be noticed that the fibers length in the final composite are 60~70 μm on average, far shorter than the raw ZrO_2 (f)_, mainly caused by worn during the ball milling. In fact, in order to achieve better reinforcement, the damage to chopped fibers should be avoided. However, the ball milling, an effective and simple dispersion approach, is widely used for dispersion of high solid loading slurry with chopped fibers, which leads to the inevitable damage to chopped fibers. Therefore, it is very important to optimize the ball milling process, especially the duration of ball milling. In this study, the duration of ball milling is controlled in 40 min, to obtain a well-dispersed slurry without the severe damage of fibers.

The primary toughening mechanism of the chopped fiber reinforced ceramic matrix composite is the fiber pulling-out, bridging, and deboning. The fiber pulling-out and bridging can be obviously observed in Figure 4a–d, and the deboning can be observed in Figure 4e. The EDS was carried out to identify the composition on the interface of fiber and CaO matrix. According to the proportion of the elements distribution, as shown in Figure 4f, it can be inferred that the main component of the interface on the fiber may be the calcium zirconate (CaZrO_3_), formed by the reaction of CaO and ZrO_2_ at elevated temperature, as shown in Equation (2). It indicates that a strong interface is formed on the surface of fibers, which could transfer the load effectively and reinforce the mechanical properties. In addition, the layer of CaZrO_3_ on the surface of ZrO_2 (f)_ fiber will also protect the internal ZrO_2 (f)_, preventing the further reaction of ZrO_2 (f)_ and the CaO matrix at an elevated temperature.

CaO + ZrO_2_ ≜ CaZrO_3_ (2)

**Figure 5:**
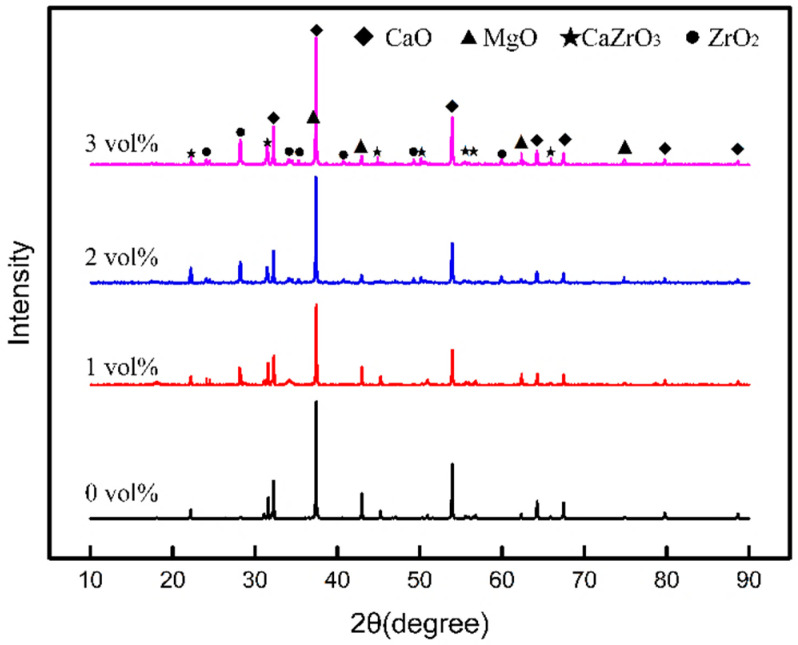
XRD Patterns of sintered ceramic mold with different ZrO_2 (f)_ content.

Figure 5 shows the XRD pattern of the sintered CaO-based ceramic mold with different content of ZrO_2 (f)_. As can be seen, these patterns are dominated by the diffraction peaks of CaO, MgO, CaZrO_3_, and ZrO_2_. The diffraction peaks of CaO and MgO should be determined by the raw materials of CaO and MgO in the ceramic matrix. The diffraction peaks of ZrO_2_ should be the characteristic of ZrO_2 (f)_, because the zirconium powder added to the matrix material is easily oxidized to ZrO_2_ when the green body is sintered in the atmosphere, then reacts with the CaO matrix to form a CaZrO_3_ phase, and there is no other source of ZrO_2_. In addition, the peak intensity of ZrO_2_ gradually increased with the increase of the content of ZrO_2__(f)_, indicating that the ZrO_2_ phase in the ceramic mold mainly comes from ZrO_2 (f)_, and the ZrO_2 (f)_ still existed as fiber, although the surface of the ZrO_2 (f)_ reacted with the CaO matrix to form a CaZrO_3_ phase, which is also consistent with the SEM results observed in the microstructure.

### 3.3. Mechanical Properties and Sintering Shrinkage

**Figure 6:**
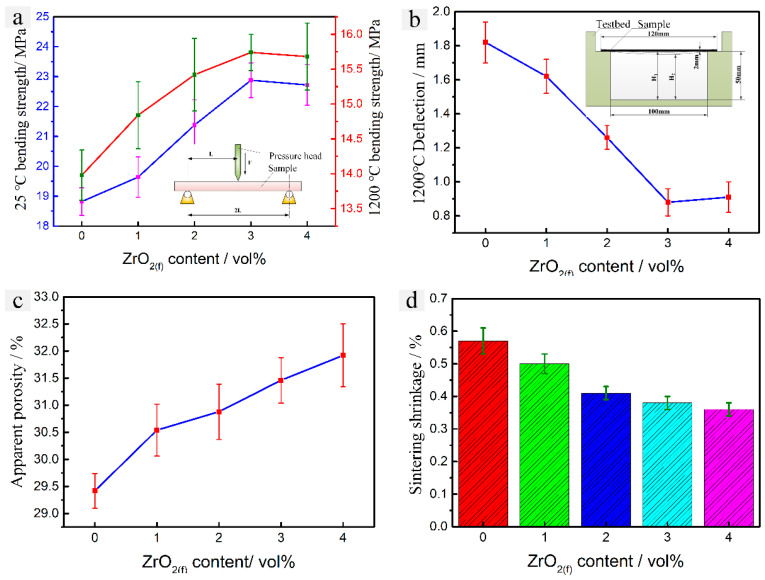
Effect of ZrO_2 (f)_ content on properties of CaO-based ceramic mold: (**a**) room and elevated temperature (1200 °C) bending strength, (**b**) deflection (1200 °C), (**c**) apparent porosity, (**d**) sintering shrinkage.

Figure 6 shows the mechanical properties and sintering shrinkage of the samples fabricated with different ZrO_2 (f)_ content after sintered. As can be seen from Figure 6a, both the room temperature (25 °C) and elevated temperature (1200 °C) bending strength increased with the ZrO_2 (f)_ content varying from 0 vol % to 3 vol % and peaked at 22.88 MPa and 15.74 MPa, respectively, when ZrO_2 (f)_ content is 3 vol %. Further increase the content of ZrO_2 (f)_ resulted in a slight decrease of bending strength due to the inhomogeneous microstructure of CaO-based ceramic sample caused by the heterogeneous distribution of excessive ZrO_2 (f)_. As can be seen from Figure 6b, the elevated temperature (1200 °C) deflection decreased as the ZrO_2 (f)_ content increased from 0 vol % to 3 vol % and the minimum deflection is 0.86 mm, when ZrO_2 (f)_ content is 3 vol %. A slight increase of deflection was exhibited with the further addition of the ZrO_2 (f)_ caused by the heterogeneous distribution of excessive ZrO_2 (f)_ as well. As can be seen from Figure 6c,d, the apparent porosity increased from 29.46% to 31.42% and the sintering shrinkage decreased from 0.57% to 0.40% as the ZrO_2 (f)_ content increased from 1vol% to 3 vol %. It indicates that the chopped ZrO_2 (f)_ may well be formed in a bracket for the CaO ceramic matrix, and the CaO ceramic particles are fixed to retard the translocation of ceramic particles, so that the porosity of the ceramic mold increased and the sintering shrinkage decreased. It showed that the mechanical properties and the sintering shrinkage could be significantly improved by the addition of ZrO_2 (f)_.

### 3.4. Case Study

**Figure 7:**
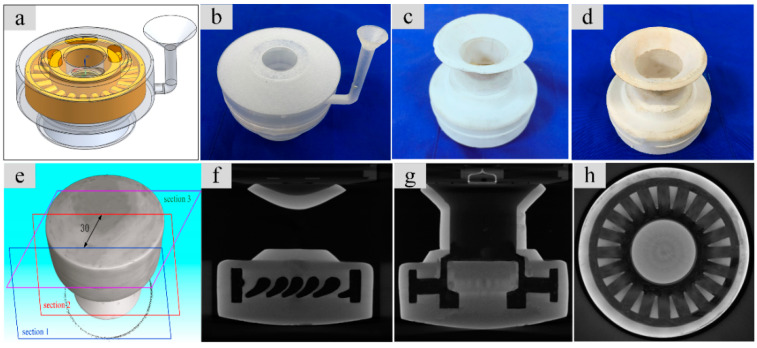
Fabrication of CaO-based ceramic mold of an impeller and the CT image of mold: (**a**) CAD mold, (**b**) resin mold, (**c**) dried green body, (**d**) sintered ceramic mold, (**e**) scan section of CT, (**f**) CT image of Section 1, (**g**) CT image of Section 2, (**h**) CT image of Section 3.

A CaO-based ceramic mold of impeller was fabricated by the RIFT with the ZrO_2 (f)_ content of 3 vol % according to the procedure described in Section 2, as shown in Figure 7a–d and the internal structure of the mold was tested by industrial CT, as shown in Figure 7e–h. X-ray analysis reveals that the shape of the impeller was exactly duplicated and the internal structure of the impeller ceramic mold was well maintained, indicating that the CaO-based ceramic mold reinforced by chopped ZrO_2 (f)_ can meet the requirements of casting.

## 4. Conclusions

A CaO-based integral ceramic mold with improved mechanical properties has been fabricated by SLA and TBA-based gel-casting with the chopped ZrO_2 (f)_ reinforced method. In order to obtain a sufficiently filled green body of CaO-based integral ceramic mold with tiny structure, the content of ZrO_2 (f)_ should not exceed 3 vol %. SEM and XRD analysis shows that the sample fabricated with the addition of ZrO_2 (f)_ content of 3 vol % has a microstructure with a homogeneous distribution of ZrO_2 (f)_, which reinforces the CaO-based ceramic mold with fiber pulling-out, bridging, and deboning. The room temperature (25 °C) bending strength increased to 22.88 MPa, elevated temperature (1200 °C) bending strength increased to 15.74 MPa, sintering shrinkage decreased to 0.40% and apparent porosity increased to 31.42%, respectively. This CaO-based ceramic mold is an excellence candidate to be used in investment casting for the aerospace and engineering fields.

## Figures and Tables

**Table 1:** Component of the composite powders.

Sample Number	Volume Fraction (vol %)			
	CaO	MgO	Zr	ZrO_2 (f)_
A	42.00	12.00	2.00	0.00
B	41.00	12.00	2.00	1.00
C	40.00	12.00	2.00	2.00
D	39.00	12.00	2.00	3.00
E	38.00	12.00	2.00	4.00
